# Dental Department University of Maryland Commencement

**Published:** 1899-04

**Authors:** 


### Editorial, Etc.

### DENTAL DEPARTMENT UNIVERSITY OF MARYLAND COMMENCEMENT.

Sixty-five young men became dentists last night at the Lyceum Theatre, and now, fortified with their fresh diplomas, strengthened by three years of hard study and enthused by the plaudits of friends and relatives, they are ready to assist in the noble work of relieving that portion of the human race which suffers with its teeth.

These sixty-five young men were the graduates of the class of 1899 of the department of dental surgery of the University of Maryland, which department celebrated its eighteenth annual commencement last night. The theatre was crowded, the boxes were filled with the mothers, sisters, sweethearts and friends of the young dentists, and two ushers were much overworked in distributing the large and beautiful bouquets sent by the admirers of the class.

The exercises were opened with prayer by Rev. Samuel A. Wilson, which was followed by the reading of the mandamus by the dean, Prof. Ferdinand J. S. Gorgas. There was some music by the orchestra, and the degrees were conferred by the dean, in the absence of Mr. Bernard Carter, provost of the University.

Rev. Dr. Elbert S. Todd, of the Strawbridge M. E. Church, made the address to the graduates. He began by emphasizing the fact that in making a dentist, as in the making of a surgeon, a lawer or a priest, there must first be a man to start with. Manhood stands over against beasthood and is a stage beyond boyhood. Character cannot be separated from professional life. In view of the fact that some of the graduates might be called into public life, Dr. Todd advised them to acquire knowledge of American institutions. The ability to get votes enough to be elected would not make up for lack of that knowledge. In conclusion Dr. Todd said the openings for professional work might seem few, but death was always making vacancies. The best opening is one developed by patient and painstaking work.

The prizes were awarded to the winners by Prof. Francis T. Miles, each fortunate graduate coming forward as his name was called.

The class oration was delivered by Linnaeus C. Shecut, of South Carolina, who is the winner of the University gold medal and attained the highes grading of the entire class.

The prize-winners were Linnaeus C. Shecut, first prize; Walter T. Lineweaver, Tamji Takashima, Hermann Tropp, John W. Carlton, Charles E. Outcalt, Samuel B. Milford, James H. Baker, William Raddin Pond. These were the senior year prizes. The junior year prizes were won by John S. Earman, B. F. Leonard, Samuel S. Burt, Joseph T. Arnold. The freshman prizes were won by J. E. Ewing, Eugene Chew, W. T. F. Hamilton.

The students graduating nearest a possible 700 were Linnaeus C. Shecut, Walter T. Lineweaver, J. N. Johnson, Andrew B. Wardlaw, Cyrus Kurtz, S. B. Moscovitz, Warren Price, Maughs M. Brown, W. L. Logan, Pearson Du B. Brooker, John R. Copeland, S. B. Milford.

Those who received honorable mention were F. E. Smith, H. B. Smith, G. C. Smith, W. R. McCleod, C. Kurtz, A. B. Wardlaw, E. V. McConnell, W. L. Logan, S. O Marshall, Warren Price, D. G. Hollinger, W. P. Scott, H. C. Stover, W. S. Brown, S. B. Milford, E. Gamard, A. I. Harris, B. F. Mann, R. W. Jackson, J. T. Arnold, B. F. Leonard, M. D., J. W. Wooton, J. S. Rockweel, E. W. Thomson.

The exercises were closed with a benediction by the Rev. Dr. Todd.

